# Identification of Plasma Biomarkers from Rheumatoid Arthritis Patients Using an Optimized Sequential Window Acquisition of All THeoretical Mass Spectra (SWATH) Proteomics Workflow

**DOI:** 10.3390/proteomes11040032

**Published:** 2023-10-16

**Authors:** Liang Jin, Fei Wang, Xue Wang, Bohdan P. Harvey, Yingtao Bi, Chenqi Hu, Baoliang Cui, Anhdao T. Darcy, John W. Maull, Ben R. Phillips, Youngjae Kim, Gary J. Jenkins, Thierry R. Sornasse, Yu Tian

**Affiliations:** 1 Research & Development, AbbVie, North Chicago, IL 60064, USA; liang.jin@abbvie.com (L.J.); bohdan.harvey@abbvie.com (B.P.H.); baoliang.cui@abbvie.com (B.C.); anhdao.darcy@abbvie.com (A.T.D.); john.maull@abbvie.com (J.W.M.); thierry.sornasse@abbvie.com (T.R.S.); 2 DMPK, Takeda Development Center Americas Inc., Cambridge, MA 02142, USA; chu8uic@gmail.com (C.H.);

**Keywords:** rheumatoid arthritis, proteomics, SWATH, DIA, plasma, biomarker, protein isoform

## Abstract

Rheumatoid arthritis (RA) is a systemic autoimmune and inflammatory disease. Plasma biomarkers are critical for understanding disease mechanisms, treatment effects, and diagnosis. Mass spectrometry-based proteomics is a powerful tool for unbiased biomarker discovery. However, plasma proteomics is significantly hampered by signal interference from high-abundance proteins, low overall protein coverage, and high levels of missing data from data-dependent acquisition (DDA). To achieve quantitative proteomics analysis for plasma samples with a balance of throughput, performance, and cost, we developed a workflow incorporating plate-based high abundance protein depletion and sample preparation, comprehensive peptide spectral library building, and data-independent acquisition (DIA) SWATH mass spectrometry-based methodology. In this study, we analyzed plasma samples from both RA patients and healthy donors. The results showed that the new workflow performance exceeded that of the current state-of-the-art depletion-based plasma proteomic platforms in terms of both data quality and proteome coverage. Proteins from biological processes related to the activation of systemic inflammation, suppression of platelet function, and loss of muscle mass were enriched and differentially expressed in RA. Some plasma proteins, particularly acute-phase reactant proteins, showed great power to distinguish between RA patients and healthy donors. Moreover, protein isoforms in the plasma were also analyzed, providing even deeper proteome coverage. This workflow can serve as a basis for further application in discovering plasma biomarkers of other diseases.

## 1. Introduction

Rheumatoid arthritis (RA) is an inflammatory autoimmune disease that can lead to significant joint destruction (cartilage/bone erosion) and muscle wasting (cachexia) if left untreated. Blood-borne factors associated with inflammation and tissue breakdown are readily detectable in patients with severe RA [1]. This has led to the development of valuable diagnostic tools, such as the C-reactive protein (CRP) test and 14-3-3 eta protein assay, which enable clinicians to make a more accurate diagnosis of RA [2]. However, with the advent of several effective, though limited, remittive therapies, the demand for more sensitive biomarker tools to discriminate and predict patients’ responses has greatly increased [3]. This demand has propelled investigations into identifying novel blood-borne biomarkers using global proteomic profiling that analyzes samples in an untargeted broad-scale approach. Among the available plasma biomarker discovery approaches, including the Proximity Extension Assay (Olink), aptamer-based (SomaScan) [4,5], and mass spec-based proteomics, only the MS-based approach is reagent-independent and truly unbiased.

Identifying biomarkers from plasma using mass spec proteomics analysis is exceptionally challenging not only in biology but also from an analytical perspective. On one hand, the concentrations of plasma proteins can span an extremely wide range of over 10 orders of magnitude (from ~80 mg/mL to pg/mL), while on the other hand, a small number of high-abundance proteins consist of over 90% plasma protein content [6]. Therefore, mass spec signal suppression from the high-abundance proteins can prevent the efficient detection of a large population of low-abundance proteins using mass spectrometry (MS)-based proteomics, which limits the proteome coverage of plasma compared to other matrixes [7]. To overcome these intrinsic issues of plasma proteomics, efforts have been made in this field on improving sample preparation: (a) depletion of high-abundance proteins [6]; (b) extensive fractionation to make low-abundance proteins detectable and utilizing advanced MS technologies, including [7]; (c) DIA (Data-Independent Acquisition) including SWATH (Sequential Window Acquisition of all THeoretical mass spectra) with fast scanning [8]; and (d) advanced PASEF (Parallel Accumulation–SErial Fragmentation technique) to further increase proteome depth and throughput, which is critical for making the workflow practical for biomarker discovery studies [9,10]. One attractive approach for plasma proteomic analysis to highlight is to integrate nanoparticle (NP) protein coronas with LC-MS [11,12]. The engineered NPs contain various physicochemical properties for nano-bio interactions, and each NP can interrogate hundreds of proteins in an unbiased manner to execute a highly parallel protein separation prior to MS. Using a 96-well automated workflow (Proteograph^TM^), a panel of five NPs detected >2000 proteins across 141 plasma samples with DIA-MS (EKSPERT nano-LC425 -Triple TOF 6600+, Framingham, MA, USA) in a non-small cell lung cancer classification study; in contrast, only >100 proteins were detected from neat plasma without any depletion or fractionation [11]. This platform leverages a rapid and deep profiling of the plasma proteome. However, it relies heavily on expensive NP products (Proteograph^TM^ XT assay kit) and automation instruments (SP100 automation, Seer, Redwood City, CA, USA), which increases the budget burden and makes it impractical for large-scale studies, especially in startup laboratories. In addition, the required plasma sample volume could be up to 250 µL, instead of the traditional <10 µL, which may not be practical for sample procurement in animals (e.g., mice) and certain clinical studies. Another recently developed platform utilizes selective plasma protein precipitation by perchloric acid (perCA) [9], resulting in the detection of >1300 proteins per run and up to 60 SPD (samples per day), with an Evosep LC connected to a timsTOF Pro, enabling DIA-PASEF. This approach has shown the high-throughput ability to process >3000 samples without an obvious batch effect and reduces the cost to ~$2.5 per sample. However, this time and cost-effective platform still requires 50 µL of plasma and a higher-end LC/MS system, such as timsTOF Pro or equivalent, which limits the sample procurements and laboratories facilities.

Compared to the platforms mentioned above, conventional MS-based proteomics is more cost effective, requires less sample volume, and is more suitable for large-scale analysis. In the plasma proteomics workflow used in this study, we combined the construction of a comprehensive experiment-specific spectral library, plate-based sample preparation including high-abundance protein depletion, plate-based mixed-mode solid-phase extraction sample cleanup, and SWATH-DIA mass spec data acquisition, followed by spectral library data matching in DIA-NN. This workflow requires only 10 µL of plasma to achieve good proteome coverage. We further optimized each step to enforce a balance between proteome coverage and throughput. As it does not rely on high-end instruments or high volumes of plasma samples, this workflow could be a practical option for large-scale plasma proteomics, especially for plasma proteomics applications with limited sample volumes.

In this study, 80 plasma samples from RA patients (*n* = 60) and healthy donors (*n* = 20) were analyzed using the optimized SWATH proteomic workflow, resulting in 663 proteins being identified and quantified on average across subjects with low missing values (7.6% for all samples, 5.2% for healthy controls). Differentially expressed plasma proteins were identified using statistical analysis, and associated pathways were determined using over-representation analysis (ORA). The random forest algorithm was used to identify proteins that could discriminate between RA and healthy plasma samples. To further evaluate the performance of this workflow, we investigated the consistency between this study and other proteomic studies with not only plasma but also synovial tissue and synovial fluid samples from RA patients.

## 2. Materials and Methods

### 2.1. Human Plasma Samples

The subset of patients included in this study was randomly selected from patients enrolled in the SELECT-COMPARE study (NCT02629159) [13] who consented to exploratory research. All patients had active RA despite treatment with methotrexate for at least three months. Only baseline plasma samples (K2-EDTA blood collection) were selected for this experiment. EDTA plasma samples from healthy volunteers were obtained from Conversant Biologics, Inc., Huntsville, Alabama, USA.

### 2.2. Sample Fractionation and Spectral Library Generation

#### 2.2.1. Sample Preparation

To prepare the experiment-specific plasma peptide spectrum library, plasma from RA patients and healthy donors was pooled. Briefly, an equal volume (30 µL) of plasma from each of the study cohorts, 60 RA patient samples and 20 healthy controls, was pooled, resulting in a total of ~2.4 mL of pooled plasma at a 3:1 *v*/*v* ratio of RA to control ratio. The rationale is to have a representation of disease vs. healthy subjects based on the distribution of the study cohort.

#### 2.2.2. Size Exclusion Chromatography (SEC) Fractionation

SEC chromatography was used as protein-level fractionation to reduce the complexity of plasma proteins before building the peptide spectral library. Pooled human plasma samples (1 mL) were filtered using a 0.2 µm membrane centrifuge device (CN# ODM02C34, PALL, Marlborough, MA, USA), and fractionated using a Superdex-200 10/300L SEC column (CN# 28990944, GE Health, Marlborough, MA, USA) on a Dionex Ultimate 3000 LC system with an Analytics SFM Sample and Fraction Manager. The flow rate was 0.9 mL/min with PBS buffer (pH7.4). Gel protein markers (CN#151-1901, Bio-Rad, Hercules, CA, USA) were used to determine protein molecular weight (MW). Peak-based fraction collection was detected using UV absorbance signals at 280 nm. Plasma samples were fractionated into four fractions with a recovery rate of approximately 70%. After the fraction collection, each fraction was concentrated using a 30 kDa molecular weight cut-off centrifugal filter (Amicon Ultra, 15 mL, CN UFC903096, MilliporeSigma, Burlington, MA, USA), and the buffer was exchanged into 25 mM Tris pH7.4 using the Amicon MWCO filter for downstream trypsin digestion and SAX fractionation.

#### 2.2.3. Strong Anion Exchange (SAX) Fractionation

Strong anion exchange cartridges were used for peptide-level fractionation. Five aliquots of the plasma SEC fraction (~1 mL for each aliquot) were digested and used for peptide-level SAX fractionation. Briefly, 10 µL 0.5 M dithiothreitol (in 25 mM Tris, pH7.4) was added to the 1 mL of SEC fractionated plasma sample and incubated at 37 °C for 30 min to reduce the plasma proteins, followed by adding 40 µL 0.5 M iodoacetamide (IAM, Sigma-Aldrich, St. Louis, MO, USA, in 25 mM Tris, pH7.4) and incubated at room temperature in the dark for 30 min. Then, 70 µg of trypsin (Trypsin gold, MS grade, Promega, Madison, WI, USA) was added to digest the plasma proteins at a final concentration of approximately 1:50 trypsin-to-protein ratio (*w*/*w*). Strong anion exchange (SAX) fractionation was performed using a Pierce Strong Anion Exchange Spin Column (Thermo Fisher Scientific, Waltham, MA, USA) following the manufacturer’s instructions. After conditioning the spin column with 20 mM Tris-HCl (pH8.0), tryptic peptide digests (3.5 mg) were loaded and eluted through the column under a centrifugal force of 500× *g*. Flow through and additional fractions were collected by washing stepwise with 20 mM Tris-HCl (pH8.0) and increasing NaCl concentrations. A total of 5 SAX fractions were collected with final concentrations of NaCl at 0.02 M, 0.1 M, 0.25 M, 0.5 M, and 1 M NaCl, respectively.

#### 2.2.4. High-pH Reverse-Phase Fractionation

The SAX fractionated peptides were further fractionated using High-pH RPLC using an XBridge C18 4.6 × 150 mm analytical column (Waters, Milford, MA, USA) at a flow rate of 1 mL/min on an Agilent 1100 HPLC system (Agilent, Santa Clara, CA, USA)The mobile phases were 10 mM ammonium formate (pH9.0) in LC-MS-grade water (phase A) and 10 mM ammonium formate (pH9.0) in 90% acetonitrile (phase B). In total, 96 fractions were collected from each of the 5 SAX fractions through a 90 min gradient and then combined into 12 fractions following a fraction concatenation strategy [14,15]. HPLC fractions were completely dried using SpeedVac (Thermo Fisher Scientific, Waltham, MA, USA) and reconstituted with 20 μL of HPLC-grade H_2_O, 2% acetonitrile, and 0.1% formic acid (FA) for LC/MS analysis.

#### 2.2.5. Top 14 High-Abundance Proteins Depleted for Peptide Spectral Library Building

A parallel fractionation strategy was performed using depleted plasma. Briefly, 1 mL of pooled human plasma was depleted using the top 14 high-abundance protein depletion protocol based on the procedure of Section 2.3.1. The depleted plasma samples were reduced by Tris(2-carboxyethyl) phosphine (TCEP), alkylated by IAM, and digested by trypsin based on the procedure in Section 2.3.1. The resulting digested peptides were fractionated using the SAX and high-pH reverse-phase fractionation workflow, as described in Section 2.2.3 and Section 2.2.4.

#### 2.2.6. Liquid Chromatography and Mass Spectrometry Analysis Using the DDA Method

An experiment-specific peptide spectral library was constructed using a data-dependent (DDA) mass spectrometry method. The detailed procedure is based on a previous study [16] with minor revisions. Briefly, digested human plasma peptide samples from the non-depleted or depleted plasma fractionation strategies were analyzed using a capillary flow LC/MS system containing a Dionex UltiMate 3000 RSLC system (Thermo Fisher Scientific, Waltham, MA, USA) coupled online to a TripleTOF^®^ 6600 hybrid tandem mass spectrometer (SCIEX, Framingham, MA, USA) interfaced with the DuoSpray ESI source. Mobile phase A was 0.1% formic acid in 2% dimethyl sulfoxide (DMSO) in deionized water, and mobile phase B was 0.1% formic acid in 2% DMSO in acetonitrile. Then, 3–4 µg of digested peptide samples after high-pH reverse-phase fractionation was spiked with iRT peptide (Biognosys, Zurich, Switzerland) samples at a ratio of iRT/sample at 1/10 (*v*/*v*). Samples were injected into an autosampler and loaded onto a PepMAP100 C18 trap column (5 μm, 100 Å, 300 µm i.d. × 5 mm, Thermo Fisher Scientific, Waltham, MA, USA) and further gradient eluted and separated on a nanoEase *m*/*z* Peptide CSH C18 column (1.7 μm, 130 Å, 300 μm × 150 mm, Waters, Milford, MA, USA) with a flow rate of 3.1 μL/min. The HPLC gradient length was 180 min and the column temperature was 50 °C. MS1 was scanned from *m*/*z* 360–1500. The top 50 precursors were selected for fragmentation in EPI mode. The product ion mass spectrometry (MS) ranged from 100 to 1800 amu in the high sensitivity mode. Precursors with charges of 2 to 5 were selected for fragmentation, with an exclusion for 30 s after one occurrence. The accumulation times of DDA analysis were 0.25 s for precursor ions and 0.15 s for production ions. The precursor ions were fragmented using rolling collision energy. The total mass spec cycle time was 7.8 s. The TripleTOF instrument was calibrated after each sample in both MS1 and MS2 modes through LC/MS injection of beta-galactosidase trypsin-digested standards (Sciex, Framingham, MA, USA).

#### 2.2.7. Peptide Spectral Library Generation

In total, 222 (165 non-depleted plasma + 57 depleted plasma) DDA files were selected from a total of 360 files to build the spectral library based on the richness of unique peptides and the spectral quality of the DDA file. DDA MS raw files were used to perform a protein database search using MaxQuant software (version 1.6.7.0, [17]) against the Uniprot-Swissprot human protein database downloaded on 12 January 2021. The search results were imported into Spectronaut version 12.0.20491.0.14754 (Biognosys) and combined into a spectral library. Search results with no unique protein groups were excluded from library generation, resulting in a final library consisting of 165 fractions from non-depleted plasma (SEC-based protein fractionation plus SAX and high-pH reverse-phase fractionation), and 57 fractions from depleted plasma (only SAX and high-pH reverse-phase fractionation). This final library, containing 21,670 precursors and 1352 protein groups, was used for library matching of SWATH-DIA data from the study samples.

#### 2.2.8. In Silico Spectral Library Generation for Protein Isoform Analysis

To obtain a comprehensive spectral library for protein isoform analyses, we performed in silico digestion of human protein libraries from UniProt (SWISS-Prot protein isoform sequences downloaded on 12 January 2021) using DIA-NN version 1.8.1.

### 2.3. Proteomics Analysis with SWATH-DIA Workflow

#### 2.3.1. Sample Preparation

The high-abundance proteins in human plasma were depleted with Top 14 Abundant Protein Depletion Resin (Cat# A36372, Thermo Scientific, Waltham, MA, USA) in a 96-well plate to improve the throughput of plasma processing. Resin (400 μL ) was added to a 3M Empore 96-Well High-Performance Extraction Disk Filter Plate (#6065), which was fitted on top of a 1 mL deep-well plate. To reduce the sample-to-sample variant during sample preparation and control the batch effect, samples from RA patients and healthy controls were randomized before plate-based sample preparation. Plasma (10 μL ) was added to the wells, which were then incubated with depletion resins for 30 min. After centrifugation (3000 rpm, 4 °C, 5 min), 100 μL lysis buffer (2.5% sodium deoxycholate (SDC), 25 mM Tris(2-carboxyethyl) phosphine (TCEP) in 250 mM Tris pH8.5) was added to the flowthrough in the 1 mL-deep well plate at the bottom of the setup and incubated at 37 °C for 45 min. Free cysteines were alkylated with 10 mM iodoacetamide (IAM) for 30 min at room temperature in the dark. Then, 2.5 μg trypsin was added for protein digestion at 37 °C overnight. To quench the digestion, 50 μL of 10% formic acid was added to the digested samples. After centrifugation at 2000× *g* for 5 min, the supernatant was further cleaned using a strong cation exchange Oasis MCX 96-Well µElution Plate (Cat # 186001830BA, Waters, Milford, MA, USA). Briefly, the plate was equilibrated with methanol (MeOH) followed by 0.1% FA in deionized water, the samples were loaded, and the flow through was collected using a positive pressure plate manifold. The plate was washed with a solvent mixture of 32% acetonitrile, 32% MeOH, and 0.1% FA in water, followed by 5% acetonitrile/0.1% FA in water. Peptides were eluted from the plate cartridge by applying freshly prepared 2% NH_4_OH (pH11) in 55% acetonitrile twice and collected in a 1 mL-deep well plate. The eluted peptides were dried and reconstituted in 20 μL of 2% MeOH with 0.1% trifluoroacetic acid (TFA) for peptide concentration UV measurement (DropQuant, PerkinElmer, Waltham, MA, USA). The injection quantity for the SWATH-MS analysis was normalized to ~3.5 µg based on the peptide concentration measurement.

#### 2.3.2. Liquid Chromatography and Mass Spectrometry Analysis for SWATH-DIA

The LC/MS instrument hardware setup was the same as that described in Section 2.2.6, with a modification to shorten the LC gradient length to enable higher throughput sample analysis. The resulting method could support a throughput of up to 16 samples/day. Briefly, a nanoEase *m*/*z* Peptide CSH C18 Column (130 Å, 1.7 µm, 300 µm × 150 mm, Waters, Milford, MA, USA) was used on a Dionex UltiMate 3000 RSLCnano System (Thermo Fisher Scientific, Waltham, MA, USA). Mobile phase A consisted of 0.1% formic acid and 2% DMSO in deionized water, and mobile phase B consisted of 0.1% formic acid and 2% DMSO in acetonitrile. A 73 min gradient method was used to separate the peptide samples from 1% to 7% of mobile phase B in the first 5 min at a flow rate of 3.1 µL/min, 7–32.5% of mobile phase B from 5 to 62 min at 3.1 µL/min, 32.5–57% B in 0.2 min at 3.1 µL/min, 57–95% B in 3.8 min at 4.0 µL/min. The column was washed with 95% B for 3 min and equilibrated with 1% B for 4 min before the next injection. For SWATH-MS acquisition, a 3.5 µg digested peptide, containing iRT retention time reference peptides (Biognosys) at an iRT/sample ratio of 1/10 (*v*/*v*), was injected and analyzed in the data-independent acquisition (DIA) mode using the SWATH method [16]. MS1 scan was performed in the range of 360–1500 *m*/*z* in the positive ion mode. A SWATH method with 200 variable precursor isolation windows was used in this study. The MS2 window was determined by MS1 data points extracted from a DDA LC/MS run of a pooled human plasma digest sample and analyzed using the “SWATH Variable Window Calculator” Excel tool provided by SCIEX (Supplemental Material). The consecutive precursor isolation windows had a 1 amu overlap. The maximum accumulation times were 33 ms for MS1 scans and 34 ms for MS2 scans, resulting in an MS cycle time of 6.6 s. The rolling collision energy was used to determine the collision energy for each window. The mass spectrometer was calibrated using LC-MS injection of beta-galactosidase trypsin digest (SCIEX) after every two sample injections to maintain mass accuracy and resolution. To reduce run-to-run variability and potential batch effects, the injection sequence of samples from RA patients and healthy controls was randomized. Instrument QC samples (HeLa digest, 0.5 µg on column) were injected during the LC/MS batch to monitor the instrument performance. (Pierce HeLa Protein Digest Standard, 88328, Thermo Fisher Scientific, Waltham, MA, USA).

#### 2.3.3. Protein Identification and Relative Quantification

Protein identification and quantification of SWATH data were performed using DIA-NN version 1.8.1 [18] with an experiment-specific spectral library from deep fractionation. Default DIA-NN settings were adopted, except match-between-runs (MBR) was enabled and protein inference was set to “Protein names (from FASTA)”. Protein abundances were extracted from the report.pg_matrix.tsv file from the output folder of the DIA-NN. Protein IDs with more than 50% missing values were removed from further analysis. Protein abundances were then log2-transformed and normalized based on sample medians using the “medianNormalization” function from the “NormalyzerDE” R package [19], and missing values were imputed using the “missForest” R package [20], which has been demonstrated as the most robust approach [21].

The same MS raw data files were analyzed with the in-silico library built based on the human isoform protein database in DIA-NN (v1.8.1) using “Isoform IDs” for protein inference, and data analysis followed the same procedure as the above-mentioned experiment-specific spectral library. An identified protein isoform should contain at least one unique peptide mapped to its unique region compared to its canonical sequence and other isoforms belong to the same gene. Therefore, a protein group containing only one protein ID was considered a protein isoform. Furthermore, non-canonical protein isoforms were determined if the corresponding UniProt accession ID contained a hyphen and a numerical suffix indicating the isoform number. The 25 identified non-canonical isoforms were manually confirmed in the UniProt database.

#### 2.3.4. Data Analysis

To determine the differentially expressed proteins (DEPs) between RA patients and healthy donors, we used a linear model-based framework implemented in the “limma” R package [22]. Proteins with nominal *p*-values less than 0.05 and a greater than 1.5-fold change in abundance were considered differentially expressed with statistical significance and dysregulated in RA. The enriched pathways in up- and down-regulated proteins were determined by over-representation analysis (ORA) using the “WebGestaltR” R package [23] against the non-redundant Gene Ontology (GO) terms of biological processes. A random forest model was implemented using the “caret” R package [24], and ROC analysis was conducted with the “pROC” R package [25].

## 3. Results

### 3.1. Development of a SWATH Proteomics Workflow for Large-Scale Plasma Sample Analysis

**Figure 1:**
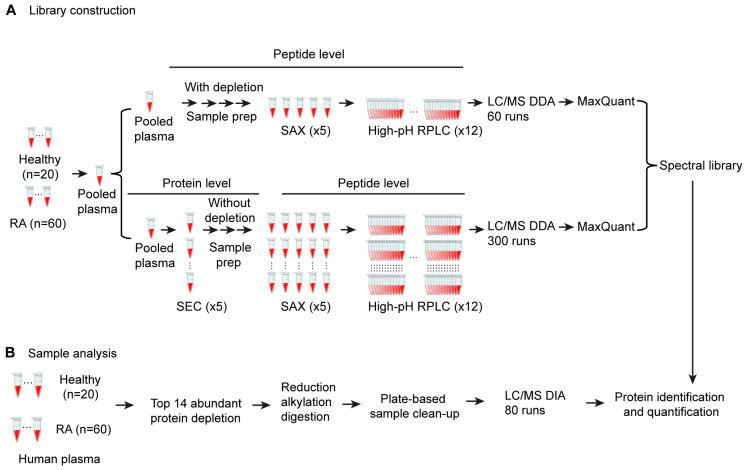
Schematic of the optimized SWATH proteomics workflow. (**A**) DDA spectral library construction procedure from pooled plasma samples. (**B**) plasma sample preparation and DIA proteomics procedure using DDA spectral library from (**A**).

The quality of the spectral library is a major factor that determines the sensitivity and depth of proteome coverage in SWATH DIA. Experiment-specific libraries generated locally have been reported to achieve better matching and quantitative performance than generic ones [26,27]. The overall proteome coverage of study samples depends on the depth of the experimental peptide spectral library. In order to maximize the coverage of the spectral library, we designed a multidimensional fractionation strategy using pooled plasma samples for library building (Figure 1). This includes a strategy of size exclusion chromatography-based (SEC) protein-level fractionation, followed by strong anion exchange (SAX) cartridge-based peptide level fractionation and high pH reverse-phase peptide-level HPLC fractionation. We also employed a parallel strategy using top 14 high-abundance protein depletion at the protein level, followed by SAX and high-pH reverse-phase peptide-level fractionation. First, five plasma protein fractions were obtained from 1 mL of pooled plasma using size exclusion chromatography (SEC) fractionation. Each fraction was trypsin digested and subjected to stepwise strong anion exchange (SAX) column elution to obtain 5 peptide fractions, each of which was further separated by high-pH reverse-phase liquid chromatography (RPLC) and fractionated into 12 concatenated fractions, resulting in 300 fractions (5 SEC × 5 SAX × 12 high-pH) in total. A second library-building set was generated by removing potential interference from high-abundance plasma proteins in LC/MS analysis through depletion of the top 14 high-abundance proteins from 1 mL of the pooled plasma sample. The depleted plasma was digested and fractionated with SAX, followed by high-pH RPLC fractionation using the same procedure as the peptide-level fractionation strategy for non-depleted plasma samples, resulting in 60 fractions. A total of 360 fractions were individually analyzed by LC/MS in DDA mode. Leveraging a database search using MaxQuant, we examined the peptides identified in each fraction and removed fractions containing no unique peptides. The search results for 165 fractions from non-depleted plasma and 57 fractions from depleted plasma were merged using Spectronaut and combined to create a peptide spectral library. This library contained 1352 protein groups, which covered a broad spectrum of plasma proteins and was used in SWATH data analysis for protein identification and quantification through library matching (Figure 1).

### 3.2. SWATH Proteomic Analysis Identified Differentially Expressed Proteins and Associated Biological Pathways in RA

**Figure 2:**
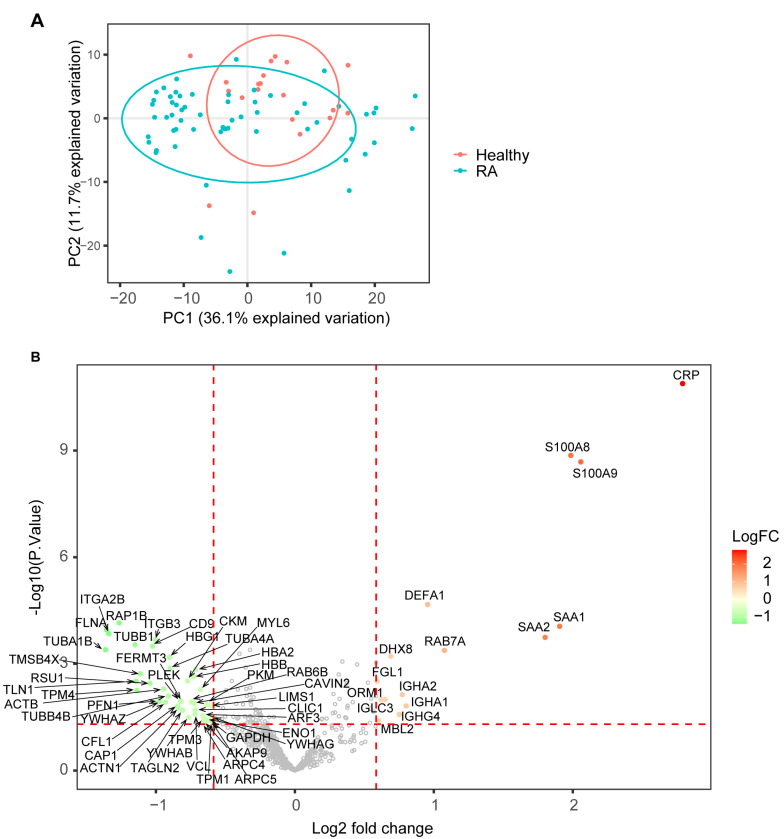
Proteomics analysis in RA and healthy groups. (**A**) principal component analysis (PCA) of plasma protein expression pattern from RA and healthy samples. (**B**) volcano plot highlighting DEPs comparing RA against healthy samples. The horizontal red dashed line indicates *p*-value = 0.05, and the vertical red dashed line indicates a 1.5-fold change in protein abundance.

To identify protein biomarkers for RA, we performed proteomic analysis of plasma samples from 20 healthy donors and 60 RA patients using the optimized SWATH platform and an experiment-specific spectral library from deep fractionation. There are 663 proteins identified and quantified after removing proteins with excessive missing data across subjects. A principal component analysis (PCA) demonstrated that the major variances of this dataset came from intra-group sample variations, as indicated by the first principal component, and patient samples had larger variations than healthy samples (Figure 2A). The second principal component was discriminative between most RA and healthy samples, whereas the clusters were not separated explicitly (Figure 2A). A differential expression (DE) analysis was performed to identify differentially expressed proteins (DEPs) in plasma samples from RA patients. In total, 58 proteins with *p*-values less than 0.05 and greater than 1.5-fold changes were considered to have statistically significant differences between RA and healthy samples, including 15 proteins increasing and 43 proteins decreasing in abundance in RA (Figure 2B, Supplementary Data S1). One of the most commonly used inflammatory biomarkers, C-reactive protein (CRP), showed the highest increase in DEP in the RA plasma. Other active inflammation-related proteins, such as serum amyloid A proteins (SAA1 and SAA2) and calprotectin (S100A8 and S100A9), also showed remarkable increases in the RA plasma (Figure 2B). Interestingly, more proteins were found to decrease in abundance in the RA plasma (Figure 2B).

**Figure 3:**
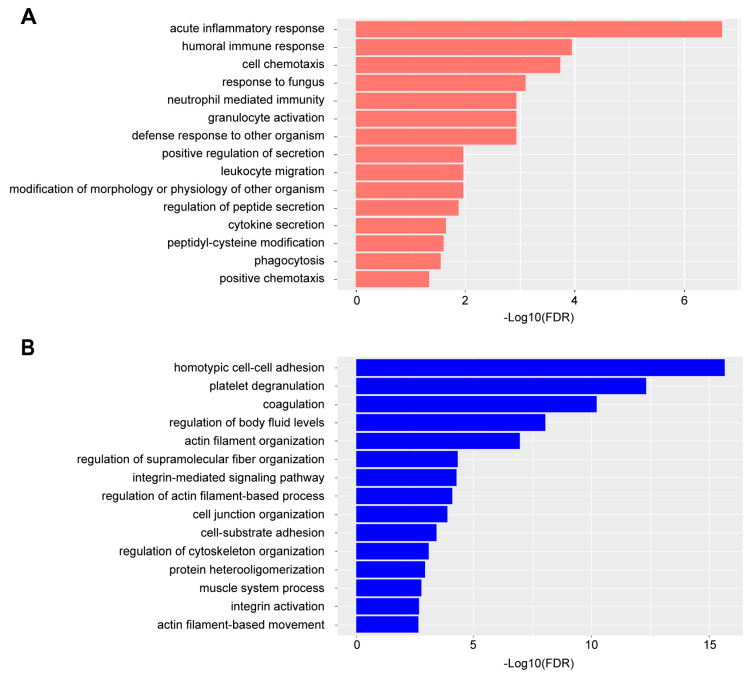
Enriched GO biological processes ORA in DEPs. (**A**) top 15 GO biological processes by in DEPs increasing in abundance ranked by false discovery rate (FDR). (**B**) top 15 GO biological processes in DEPs decreasing in abundance ranked by FDR.

To further investigate the biological mechanisms related to the DEPs in RA plasma, we analyzed the enriched biological processes in terms of Gene Ontology (GO) using an over-representation analysis (ORA). The plasma DEPs increasing in abundance in RA primarily represented signals from active inflammation and immune response, such as acute inflammatory response and humoral immune response (Figure 3A, Supplementary Data S2). Particularly, neutrophil-mediated immunity was enriched in these DEPs. A diagnostic tool that has recently been investigated for RA evaluates the neutrophil-to-lymphocyte cell count in the blood of patients. Several studies using this diagnostic tool have demonstrated that the ratio is much higher in patients with RA as compared to healthy individuals [28,29]. Upon assessment of those DEPs up-regulated in RA samples from our studies, we found that many are known to be produced by neutrophils (S100A9, S100A8, RAB7A, DEFA1, HP, LRG1, FGB, CTSG, MPO, LTF, PGLYRP1, SAA2, ORM1, ORM2, MMP9, SERPINA1, TNC, TIMP1, and PSMA6), and several have been classified as neutrophil activation markers, including calprotectin (S100A8 and S100A9 [30] HP [31], LRG1 [32], CTSG [33], LTF [34], PGLYRP1 [35], MPO [36], and MMP9 [37]). Investigations into the direct correlations between these neutrophil activation markers in blood and the circulating frequency of neutrophils are needed to corroborate these findings. On the other hand, biological processes related to platelet functions, coagulation, and the muscle system were found to be enriched in DEPs, decreasing in abundance in RA plasma samples (Figure 3B, Supplementary Data S3). Platelets have important immune effector functions in RA, and related signaling pathways are dysregulated in the presence of pro-inflammatory molecules, such as collagen, thrombin, fibrinogen, and cytokines [38]. Down-regulation of the muscle system could be related to the loss of muscle mass, which was commonly observed in the joints of RA patients [39].

Protein isoforms in the plasma have been reported as potential biomarkers for certain diseases [40,41], but such studies are rarely found in RA. Therefore, we examined the same raw DIA MS files using an in-silico-digested human protein database containing isoform sequences and focused on protein isoform detection. Twenty-five non-canonical protein isoforms were identified, with at least one unique peptide differentiating them from other isoforms. Specifically, the protein isoforms from FN1 (P02751-11), TPM3 (P06753-2), TPM1 (P09493-5), and NME2 (P22392-2) displayed a significant decrease in abundance in RA (*p* < 0.05) and a >1.5-fold decrease (Supplementary Data S4). A previous study demonstrated that the splicing machinery is impaired in RA leukocytes from peripheral blood and synovial fluid [42]; thus, the alteration of protein isoform abundance may reflect the dysfunction of alternative splicing. Interestingly, anti-TNF treatment could restore the function of the splicing machinery [42], suggesting that the protein isoforms could be good biomarker candidates to indicate the efficacy of anti-TNF treatment. Nonetheless, further investigations are needed to elucidate the biological relevance of these differentially expressed plasma protein isoforms in RA pathogenesis.

### 3.3. Meta-Analysis to Compare This Study with Other RA Omics Studies

To evaluate the findings from this study and the potential of differentially expressed proteins as RA biomarkers, a literature survey was performed using MS-based proteomic studies of serum/plasma, synovial fluid, and synovial tissue from RA patients. RNA sequencing data from the synovial tissues were also included. High-confidence biomarkers are preferred to have an alignment between systemic circulation and the disease site of action, instead of from circulation alone. The inclusion of synovial tissue and fluid proteomics could infer whether the differential expression of plasma proteins reflects the changes in RA patients’ disease tissue. Only reports within the last decade were considered, and two additional criteria were applied to filter the studies: first, comparisons between RA and control were available; second, the full lists of differentially expressed proteins were accessible, as re-analyzing previous works using raw data was not the primary intention of this work. There were limited numbers of proteomic studies of synovial tissue or fluid, including RA and healthy samples; thus, it was also acceptable if osteoarthritis (OA) was considered as the control in the selected studies. OA is a degenerative disease of local joints with a much lower inflammation level than RA and is usually used as a control in RA studies [43]. In total, we included nine reference proteomics datasets: four from serum/plasma [44,45,46,47], two from synovial tissue [48,49], and three from synovial fluid [50,51,52]. We also included another two transcriptomics datasets from synovial tissue [53,54]. Table 1 summarizes the number of reference datasets with the same common DEP increase in abundance in our dataset. The results showed that DEPs with the most significant increase in abundance, such as CRP, S100A9, S100A8, SAA1, and SAA2, were consistent with previous reports. Specifically, FGL1 was proposed as a novel and specific biomarker that could be clinically useful for predicting the progression of RA [46], which is also present in the DEP list from this study. In multi-omics studies using matching tissues, the mRNA level and protein expression normally have moderate correlations, with even lower correlations for the circulating protein expression level. Not surprisingly, there was a limited overlap in DEPs between this plasma proteomic study and previous synovial tissue transcriptomic datasets, indicating the distal nature of circulating protein biomarkers in the plasma of RA patients. Nonetheless, activated neutrophils have been detected in high numbers in the synovial joints and tissues of RA patients [55,56], and upon comparison with the transcriptomic profile of RA synovial fluid neutrophils [57], several of the proteins identified in the plasma of RA donors as potentially attributable to activated neutrophils were found to be expressed in this pro-inflammatory neutrophil population, including MMP9, ORM1, ORM2, S100A8, S100A9, and TKT (Supplementary Data S1).

### 3.4. Biomarker Identification Using Random Forest to Discriminate between RA and Healthy Plasma

**Figure 4:**
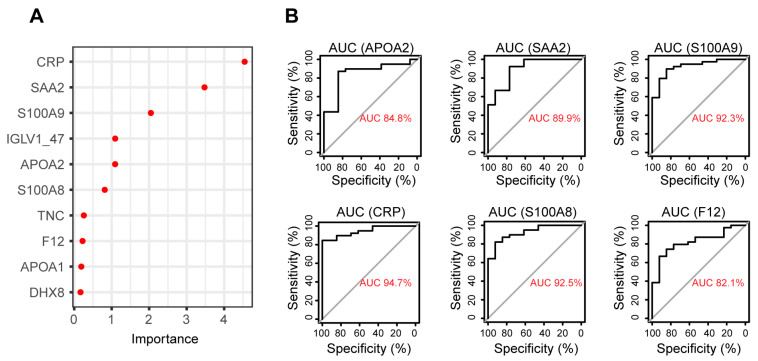
Biomarker discovery by random forest. (**A**) top 10 proteins with highest feature importance in determining model accuracy. (**B**) ROC plots of proteins with AUC greater than 0.8 in distinguishing RA from healthy samples.

In recent years, machine learning algorithms have been widely used in biomarker discovery for sample classification and feature selection. In this study, 70% of the samples were randomly selected to train a random forest (RF) model with a 5-fold cross-validation to distinguish RA from healthy samples. The model was validated by predicting the remaining 30% of the samples and achieved a classification accuracy of 82%. The proteins that accounted for the highest importance of model accuracy were CRP, SAA2, S100A9, IGLV1-47, APOA2, S100A8, TNC, F12, APOA1, and FHX8, which could be considered potential biomarkers (Figure 4A). The area under the curve (AUC) was also calculated for the receiver operating characteristic (ROC) analysis to evaluate the discrimination performance of these proteins. Six proteins achieved AUC greater than 0.8, including CRP, SAA2, S100A9, APOA2, S100A8, and F12 (Figure 4B), indicating that these proteins could have great power to discriminate between RA and healthy samples based on plasma protein expression. However, these proteins have been reported to be associated with inflammation and used as biomarkers for other inflammatory diseases such as inflammatory bowel disease (IBD) [58]. Therefore, these proteins may not be specific enough to the disease, which limits their application as RA-specific plasma biomarkers.

## 4. Discussion

In this study, we analyzed 80 plasma samples from RA patients and healthy donors using an optimized SWATH DIA proteomics strategy. The comprehensive experiment-specific spectral library from deeply fractionated pooled samples, plate-based sample preparation procedure, and robust LC-MS system enabled high-throughput deep proteome coverage. Among the 663 proteins identified and quantified, 58 were found differentially expressed in RA with statistical significance. The DEPs that were increased in RA were mostly linked to active inflammation and immune response, and DEPs with decreased abundance in RA presented platelet and coagulation dysfunction and a reduction in muscle mass. Our results, particularly the increase in DEPs in RA, showed explicit agreement with other recent plasma proteomics studies. Many of the DEPs that increased in abundance in RA plasma were also found to be more abundant in RA synovial fluid and synovial tissue, indicating that these plasma proteins could reflect the disease-related alterations in protein expression in the joints of RA patients. A random forest model trained with this dataset was able to discriminate RA samples from healthy samples with an accuracy of 83%, and the proteins with the highest importance to the model accuracy also showed great discriminative power according to the ROC analysis. This study provides a feasible option for implementing high-throughput SWATH-DIA proteomics technology to analyze plasma samples and generate high-quality quantitative data with good proteome coverage.

In this SWATH-DIA workflow, the proteome coverage was considerably higher than that of traditional DDA analysis. Compared with the general average of 200–500 plasma proteins identified [45,47,59,60], 663 proteins were identified in this study, indicating a remarkable increase in the depth of proteome coverage. Several steps were taken to improve proteomics analysis sensitivity and data quality: 1. DMSO as an additive to the HPLC mobile phase [16,61], 2. Capillary flow HPLC method [62]. The flow rate range of 3–5 µL/min is a sweet spot where the system can still fit a regular ESI source without a true nano ESI source, while the system can still benefit from sensitivity improvement at a lower flow rate. In contrast, at a high flow rate of 20–50 µL/min, the sensitivity approaches a regular analytical flow LC/MS without the benefit of low flow rate sensitivity improvement; 3. Variable window SWATH method. Because the peptide abundance across the *m*/*z* range is not evenly distributed for trypsin-digested samples, an evenly distributed SWATH window will result in overcrowding of peptides in certain ranges, leading to complex chimeric spectra and lower library match data quality, while under-sampling in other *m*/*z* ranges with fewer abundant peptides. To increase the specificity of the DIA MS2 analysis and avoid the uneven distribution of MS2 resources, a variable SWATH window strategy was used. Based on TripleTOF vendor specifications, 200 is the maximum allowable window size. However, to accommodate all 200 windows without exceeding the allowable MS cycle time, careful balancing of the window size, accumulation time, and overall MS cycle time is needed. Attempts to increase the flow rate, sharpen the peak shape, and maximize the number of data points per peak were made. The final method has a total MS cycle time of 6.7 s which can support 7–8 data points per HPLC peak as a balance of sensitivity, proteomics coverage, robustness, and quantitative performance without sacrificing system stability and robustness.

Plasma is a complex biological matrix. There are multiple challenges in large-scale plasma proteomics sample preparation: 1. high-abundance plasma proteins and large dynamic range of plasma proteins concentrations (>12 orders of magnitude) [6]; 2. high concentrations of lipids (~5 mg/mL) [63]; 3. sample cleanup is critical after reduction and alkylation before LC/MS analysis. To achieve reproducible and robust plasma proteomics analysis, we adopted a plate-based workflow to address these challenges, which include: 1. plate-based top 14 high-abundance protein depletion; 2. mixed mode solid-phase-extraction plate-based desalting and sample clean-up. In the field of mass spec proteomics, most of the studies were conducted using nanoflow HPLC to achieve maximum sensitivity. However, there are multiple disadvantages to using nano-LC platforms, such as long LC gradients, slower sample loading time, lower robustness, shorter column life, etc. The capillary HPLC system was exceptionally suitable for the Sciex TripleTOF system [16]. The column life can exceed 500 injections (internal data), which is highly desirable for complex sample types such as human plasma with large cohort sizes.

More importantly, the run-to-run consistency of the current platform is worth considering. The proteome coverage of a cohort study typically consists of three metrics: 1. the overall proteome coverage from all subjects in a study, which is the highest protein ID due to the stochastic nature of the mass spec proteomics analysis; 2. the average protein ID for each subject: 3. the overlapping protein ID across all subjects in the study, which is the lowest because of missing values across the cohort. In a quantitative proteomics-based biomarker discovery, the most valuable metric is the overlapping protein ID, which indicates consistent detection and quantitation across samples and groups. Many plasma proteomics studies have focused on the total coverage from the entire study but overlooked the coverage variability across samples [44], with a dramatic difference observed between total protein ID and overlapping protein ID. Therefore, the total protein ID is often misleading. The current platform not only generates good average proteome coverage but also contains exceptionally low missing values, making biomarker discovery more reliable.

For small-quantity proteomics applications such as single-cell or laser capture microdissection, the SWATH-DIA platform is not suitable; however, for applications where sample quality is not limited, such as plasma sample analysis, this platform is appropriate with good reproducibility and low cost. In comparison to other DIA platforms (timsTOF and Orbitrap), the current capillary flow TripleTOF SWATH-DIA platform has similar proteome coverage for plasma proteomics analysis with a similar starting volume of plasma samples. However, the peptide mass spec injection quantity requirement (3–4 µg) is approximately 5× higher than orbitrap (0.5–1 µg) and 10× higher than that of timsTOF (0.3 µg) mainly due to the lack of true nanoflow and the lack of trapping function of the TripleTOF instrument. While the overall yield of plasma proteomics sample preparation is more than sufficient to support the 4 µg injections, a higher injection quantity may lead to faster instrument contamination and performance deterioration, which should be considered for large-scale cohort analysis. To this end, the entire workflow can be easily transferred (including sample preparation and data analysis) when the mass spectrometry instrument itself is upgraded to more sensitive options.

The comprehensiveness of the spectral library is a major factor for protein detection in DIA-based proteomics. In this study, the project-specific spectral library derived from 222 fractions presented a deep coverage of plasma proteins and contributed to the identification of 663 proteins. The generation of a spectral library from deep fractionations can be time consuming, cost effective, and limited by the availability of extra samples from the same study. Nonetheless, it is beneficial for future studies with the same sample type as the spectral library. On the other hand, generating spectral libraries from in silico digestion of the FASTA protein databases has recently become increasingly popular and demonstrated equivalent performance to the DDA libraries [18,64]. We also evaluated the in silico spectral library generated by DIA-NN and it achieved overall similar proteome coverage as the DDA library, suggesting that the in-silico library could be a viable alternative if the experiment-specific library is unfeasible. However, the number of proteins identified in the in-silico library varied significantly between individual samples. Interestingly, fewer proteins were identified using the merged DDA and in silico libraries; thus, merging the DDA and in silico libraries is not pursued for the current study. Based on this learning, the effort of protein isoform analysis was entirely based on in silico libraries without rebuilding peptide spectral libraries based on DDA runs. Available alternative splicing isoform analyses using mass spectrometry proteomics from human plasma are limited. The fact that the current platform can analyze splice variants exemplifies its unique capabilities.

The latest build of the Human Plasma Proteome Project has 4395 canonical proteins [65], with an average of ~450 proteins from each experiment and even lower protein coverage per sample. The current paper presents the advancement of shotgun plasma proteomics (bottom-up approach) by using a comprehensive peptide library and DIA approach. However, the shotgun approach still only covers a narrow range of protein sequences (1–2 peptides) for the low-abundance proteins. Although only one unique peptide per protein was sufficient for protein inference from peptide-spectral matching (PSM), the detected peptides did not necessarily contain unique isoform sequences. On the other hand, the possible number of proteoforms in human plasma is much higher [66,67].

Proteoforms are the different forms of a protein with a variety of sequence variations, splice isoforms, and post-translational modifications [68,69]. One of the most important sources of proteoform complexity is alternative splicing. Some genes can produce multiple isoforms of the same protein through alternative splicing or transcription initiation, which might have distinct functions and properties. Differential protein expressions related to disease may not be manifested at the overall protein expression level but at the splice variant isoform level [70]. Although trypsin digestion-based shotgun proteomics provides high coverage of canonical protein sequences, the detection and quantitation of splice variants using shotgun proteomics is limited as the unique sequences of splice variants are often masked by the trypsin digestion site [71]. Protein post-translational modification is another source of proteoform complexity in disease states [72]. The most common PTM in plasma proteins is glycosylation [73], while the measurement of glycopeptides using the shotgun proteomics method is a specialty by itself, with dedicated sample preparation, chromatography, and mass spec fragmentation mechanisms [73,74].

An intact analysis of plasma proteins is more appropriate to investigate disease-related proteoforms. However, the analysis and quantification of proteoforms at the intact level is highly challenging. Among the different analytical options, highly sensitive reagent-based assays, such as SomaScan or Olink, require the development of proteoform-specific reagents (aptamers or antibodies) for each proteoform. While mass spectrometry is highly capable of analyzing proteoforms and there has been tremendous progress in the field of top-down proteomics [75], the coverage of top-down proteomics analysis is still too low to be used as the primary biomarker discovery tool [73]. Most of the advances are using top-down mass spectrometry to study low-molecule-weight proteoform species [76] or based on sample fractionation [77] or immunoaffinity sample enrichment of the targeted proteoform analysis [78]. Another limitation of intact proteoform analysis is for glycoproteins, as the complexity of glycoforms could be overwhelming [79].

One possible strategy could be using shotgun proteomics results to nominate proteoform biomarker candidates, for example, using shotgun proteomics to identify splice variants [80]. The shortlist of isoform biomarker candidates could be validated by using antibody-based immunoaffinity enrichment, followed by targeted intact protein/top-down MS analysis [78]. The glycoprotein proteoform can be analyzed using shotgun glycoproteomics [73] or intact protein analysis for purified proteins [81]. To achieve higher splice variance in protein isoform detectability, the proteomics platform needs to have good overall protein sequence coverage and peptide level coverage. The current study provides an example of improved peptide-level proteome coverage. One explanation is that the current platform is based on an in-depth peptide-spectral library building, which helps provide a large splice isoform-specific peptide repertoire. There are still many limitations to this approach, but it provides a starting point for the comprehensive proteoform biomarker discovery process.

In addition to the technical improvements in workflow and detection sensitivity, the current study also highlights that these modifications can be implemented without sacrificing the ability to detect biologically meaningful differences in the proteomic profile of plasma from RA patients compared to healthy donors. A differential expression analysis and pathway over-representative analysis revealed that the change in protein expression profiles in RA plasma primarily reflected active inflammation and immune response, as well as the repression of platelet functions (Figure 2 and Figure 3). Some of the DEPs that increased in abundance in the plasma may indicate enhanced expression of the same proteins in synovial tissues and fluids of RA patients (Table 1). Particularly, the expression of calprotectin (S100A8 and S100A9) showed the capability to discriminate between RA and healthy plasma samples (Figure 4). However, most of the upregulated proteins detected in this study were typical inflammatory markers that are not specific to RA, thus undermining their value as diagnostic biomarkers for RA. For example, inflammatory bowel disease (IBD), which includes Crohn’s disease and ulcerative colitis, is a chronic inflammatory disorder of the gastrointestinal tract. Studies have reported that C-reactive protein, serum amyloid A, and calprotectin all show diagnostic value for IBD with high sensitivities and specificities [82,83,84,85]. Therefore, non-inflammatory RA-specific biomarkers require further investigation. A comparison of protein expression profiles between RA and other inflammatory disorders, such as asthma and IBD, could potentially lead to more specific biomarker discovery for RA [58]. Despite this, these proteins can potentially be used as generic indicators of treatment effectiveness for these inflammatory diseases.

Besides the canonical proteins, this study identified 25 non-canonical protein isoforms from human plasma samples and 4 of these, FN1 (P02751-11), TPM3 (P06753-2), TPM1 (P09493-5), and NME2 (P22392-2), showed greater than 1.5-fold change decreases in RA with statistical significance (Supplementary Data S4). Fibronectin (FN1) has 15 isoforms from alternative splicing, and different isoforms have been reported to be present in synovial fluid from RA patients [86]. Interestingly, fibronectin has been found to promote differentiation and mineralization of osteoblasts [87], thus the decrease in fibronectin isoforms in RA plasma may indicate dysfunction of osteogenesis. Further investigation of the isoforms dysregulated in RA could reveal more biological mechanisms.

In addition to LC/MS-based proteomics, capture reagent-based proteomics platforms, such as OLINK and SomaScan, are popular options for analyzing plasma proteins for biomarker discovery. These platforms provide a wide range of options for quantifying hundreds to thousands of plasma proteins from a single sample and cover broad dynamic ranges to enable the detection of low-abundant proteins [4,5,88]. Compared to these platforms, LC/MS proteomics is not limited by the availability of detection reagents but lacks the sensitivity to identify low-abundance proteins. Therefore, LC-MS and capture reagent-based proteomics platforms are complementary. Mass spec-based plasma proteomics has witnessed tremendous progress in recent years through the development of deep fractionation and efficient depletion. However, nanoparticle-based protein enrichment has been the most impactful development based on nanoparticle-based protein enrichment [12]. Based on a panel of five nanoparticle types, the Seer platform can routinely analyze >1500 protein groups in human plasma using 250 µL of samples. Continuous improvements in sensitivity and throughput in mass spectrometry will enable routine analysis of human plasma at >2000 protein groups [81,89]. However, considering the cost and volume requirements of the Proteograph platform and other equivalent platforms, the approach presented here still has good value and suitability for plasma biomarker discovery in certain applications, especially for mouse plasma biomarker discovery applications where the plasma volume is limited and the suitability of the nanoparticle panel for mouse protein is still untested.

Although the platform presented here was developed for human plasma, it can be readily deployed for other matrixes for biomarker applications. Matrixes other than plasma are actually less challenging and the sample preparation procedure can be further simplified. An attractive biomarker discovery and validation approach could be using the high-throughput proteomics platform presented in this study to quickly generate biomarker “hits” using secreted matrixes, such as plasma, CSF, and feces, together with tissue of “site-of-action” and confirmed using targeted protein quantitation assays, for example, LC/MS or ligand binding assays in the secreted matrices through a more comprehensive study design. The benefit of this approach is its low cost, independence of reagent availability, and broad applicability in various matrices.

## 5. Conclusions

In summary, we analyzed plasma samples from RA patients and healthy donors using an improved SWATH-DIA proteomics workflow. A good proteome coverage of 663 proteins was achieved with low missing values. The change in protein expression in RA plasma mostly reflected active inflammation and immune response, as well as inhibition of platelet activities and loss of muscle mass. Several differently expressed proteins were found to be consistent with previous reports. Compared to the workflows that require high costs either on assays (NPs) or LC-MS instruments (Evosep LC, timsTOF), the current workflow is a viable option for plasma proteomics with satisfactory throughput and proteome coverage to enable biomarker discovery applications with cost effectiveness, and it can be easily expanded to plasma proteomic studies for other species and disease indications without sample volume restrictions.

## Figures and Tables

**Table 1:** List of common DEPs increasing in abundance between the current proteomics dataset and other previous proteomics and transcriptomics datasets.

Gene Symbol	Serum/Plasma Proteomics (4)	Synovial Tissue Proteomics * (2)	Synovial Fluid Proteomics ^#^ (3)	Synovial Tissue Transcriptomics (2)
**CRP**	3	1	0	0
**S100A9**	0	2	1	1
**S100A8**	0	2	2	1
**SAA1**	4	0	0	0
**SAA2**	2	0	0	0
**RAB7A**	1	0	0	0
**DEFA1**	0	2	1	0
**IGHA1**	0	0	1	0
**ORM1**	1	1	0	0
**FGL1**	1	0	0	0
**APCS**	3	0	0	0
**MMP9**	0	1	1	1
**ORM2**	1	0	0	0

Number in the paratheses is the number of selected papers in the corresponding category, and numbers in the table indicate the number of studies that show consistent results compared with the present study. * Comparisons between synovial tissue from RA and OA patients. ^#^ Comparisons between synovial fluid from RA and OA patients or RA and SpA patients.

## Data Availability

The MS raw files from the SWATH analysis and the project-specific spectral library are available on ProteomeXchange Consortium via the PRIDE repository (https://www.ebi.ac.uk/pride/, accessed on 15 August 2023) with the identifier PXD045171. SWATH Variable Window Calculator: https://sciex.com/form-pages/sw-downloads-form?d=SWATH-Variable-Window-Calculator.zip&asset=software&softwareProduct=SWATH%20Variable%20Window%20Calculator%201.2, accessed on 15 August 2023.

## Supplementary Materials

The following supporting information can be downloaded at: https://www.mdpi.com/article/10.3390/proteomes11040032/s1. A Supplementary Data file is available with the following information. Supplementary Data S1: Result of differential expression analysis of all proteins comparing RA vs. Healthy plasma samples. Supplementary Data S2: List of over-representative Gene Ontology Biological Processes among DEPs increasing in abundance in RA (*p*-value < 0.05). Supplementary Data S3: List of over-representative Gene Ontology Biological Processes among DEPs decreasing in abundance in RA (*p*-value < 0.05). Supplementary Data S4: Differential expression analysis results of twenty-five protein isoforms.

## References

[1] Park Y.-J., Chung M.K., Hwang D., Kim W.-U. (2015). Proteomics in Rheumatoid Arthritis Research. Immune Netw..

[2] Salman E., Çetiner S., Boral B., Kibar F., Erken E., Ersözlü E.D., Badak S.Ö., Salman R.B., Sertdemir Y., Duran A.Ç. (2019). Importance of 14-3-3eta, Anti-CarP, and Anti-Sa in the Diagnosis of Seronegative Rheumatoid Arthritis. Turk. J. Méd. Sci..

[3] Tenstad H.B., Nilsson A.C., Dellgren C.D., Lindegaard H.M., Rubin K.H., Lillevang S.T. (2020). Use and Utility of Serologic Tests for Rheumatoid Arthritis in Primary Care. Dan. Méd. J..

[4] Gold L., Ayers D., Bertino J., Bock C., Bock A., Brody E.N., Carter J., Dalby A.B., Eaton B.E., Fitzwater T. (2010). Aptamer-Based Multiplexed Proteomic Technology for Biomarker Discovery. PLoS ONE.

[5] Assarsson E., Lundberg M., Holmquist G., Björkesten J., Thorsen S.B., Ekman D., Eriksson A., Dickens E.R., Ohlsson S., Edfeldt G. (2014). Homogenous 96-Plex PEA Immunoassay Exhibiting High Sensitivity, Specificity, and Excellent Scalability. PLoS ONE.

[6] Anderson N.L., Anderson N.G. (2002). The Human Plasma Proteome History, Character, and Diagnostic Prospects. Mol. Cell. Proteom..

[7] Geyer P.E., Holdt L.M., Teupser D., Mann M. (2017). Revisiting Biomarker Discovery by Plasma Proteomics. Mol. Syst. Biol..

[8] Messner C.B., Demichev V., Bloomfield N., Yu J.S.L., White M., Kreidl M., Egger A.-S., Freiwald A., Ivosev G., Wasim F. (2021). Ultra-Fast Proteomics with Scanning SWATH. Nat. Biotechnol..

[9] Viode A., van Zalm P., Smolen K.K., Fatou B., Stevenson D., Jha M., Levy O., Steen J., Steen H., Network O. (2023). behalf of the I. A Simple, Time- and Cost-Effective, High-Throughput Depletion Strategy for Deep Plasma Proteomics. Sci. Adv..

[10] Soni R.K. (2022). High-Throughput Plasma Proteomic Profiling. Methods Mol. Biol..

[11] Blume J.E., Manning W.C., Troiano G., Hornburg D., Figa M., Hesterberg L., Platt T.L., Zhao X., Cuaresma R.A., Everley P.A. (2020). Rapid, Deep and Precise Profiling of the Plasma Proteome with Multi-Nanoparticle Protein Corona. Nat. Commun..

[12] Ferdosi S., Tangeysh B., Brown T.R., Everley P.A., Figa M., McLean M., Elgierari E.M., Zhao X., Garcia V.J., Wang T. (2022). Engineered Nanoparticles Enable Deep Proteomics Studies at Scale by Leveraging Tunable Nano–Bio Interactions. Proc. Natl. Acad. Sci. USA.

[13] Fleischmann R., Pangan A.L., Song I., Mysler E., Bessette L., Peterfy C., Durez P., Ostor A.J., Li Y., Zhou Y. (2019). Upadacitinib Versus Placebo or Adalimumab in Patients with Rheumatoid Arthritis and an Inadequate Response to Methotrexate: Results of a Phase III, Double-Blind, Randomized Controlled Trial. Arthritis Rheumatol..

[14] Yang F., Shen Y., Camp D.G., Smith R.D. (2012). High-PH Reversed-Phase Chromatography with Fraction Concatenation for 2D Proteomic Analysis. Expert Rev. Proteom..

[15] Wang H., Sun S., Zhang Y., Chen S., Liu P., Liu B. (2015). An Off-Line High PH Reversed-Phase Fractionation and Nano-Liquid Chromatography–Mass Spectrometry Method for Global Proteomic Profiling of Cell Lines. J. Chromatogr. B.

[16] Wang X., Jin L., Hu C., Shen S., Qian S., Ma M., Zhu X., Li F., Wang J., Tian Y. (2021). Ultra-High-Resolution IonStar Strategy Enhancing Accuracy and Precision of MS1-Based Proteomics and an Extensive Comparison with State-of-the-Art SWATH-MS in Large-Cohort Quantification. Anal. Chem..

[17] Cox J., Mann M. (2008). MaxQuant Enables High Peptide Identification Rates, Individualized p.p.b.-Range Mass Accuracies and Proteome-Wide Protein Quantification. Nat. Biotechnol..

[18] Demichev V., Messner C.B., Vernardis S.I., Lilley K.S., Ralser M. (2020). DIA-NN: Neural Networks and Interference Correction Enable Deep Proteome Coverage in High Throughput. Nat. Methods.

[19] Willforss J., Chawade A., Levander F. (2019). NormalyzerDE: Online Tool for Improved Normalization of Omics Expression Data and High-Sensitivity Differential Expression Analysis. J. Proteome Res..

[20] Stekhoven D.J., Bühlmann P. (2012). MissForest—Non-Parametric Missing Value Imputation for Mixed-Type Data. Bioinformatics.

[21] Jin L., Bi Y., Hu C., Qu J., Shen S., Wang X., Tian Y. (2021). A Comparative Study of Evaluating Missing Value Imputation Methods in Label-Free Proteomics. Sci. Rep..

[22] Ritchie M.E., Phipson B., Wu D., Hu Y., Law C.W., Shi W., Smyth G.K. (2015). Limma Powers Differential Expression Analyses for RNA-Sequencing and Microarray Studies. Nucleic Acids Res..

[23] Liao Y., Smyth G.K., Shi W. (2019). The R Package Rsubread Is Easier, Faster, Cheaper and Better for Alignment and Quantification of RNA Sequencing Reads. Nucleic Acids Res..

[24] Kuhn M. (2008). Building Predictive Models in R Using the Caret Package. J. Stat. Softw..

[25] Robin X., Turck N., Hainard A., Tiberti N., Lisacek F., Sanchez J.-C., Müller M. (2011). PROC: An Open-Source Package for R and S+ to Analyze and Compare ROC Curves. BMC Bioinform..

[26] Govaert E., Steendam K., Willems S., Vossaert L., Dhaenens M., Deforce D. (2017). Comparison of Fractionation Proteomics for Local SWATH Library Building. Proteomics.

[27] Anjo S.I., Santa C., Manadas B. (2017). SWATH-MS as a Tool for Biomarker Discovery: From Basic Research to Clinical Applications. Proteomics.

[28] Erre G.L., Paliogiannis P., Castagna F., Mangoni A.A., Carru C., Passiu G., Zinellu A. (2019). Meta-analysis of Neutrophil-to-lymphocyte and Platelet-to-lymphocyte Ratio in Rheumatoid Arthritis. Eur. J. Clin. Investig..

[29] Jin Z., Cai G., Zhang P., Li X., Yao S., Zhuang L., Ren M., Wang Q., Yu X. (2021). The Value of the Neutrophil-to-lymphocyte Ratio and Platelet-to-lymphocyte Ratio as Complementary Diagnostic Tools in the Diagnosis of Rheumatoid Arthritis: A Multicenter Retrospective Study. J. Clin. Lab. Anal..

[30] De Guadiana-Romualdo L.G., Rojas C.R., Morell-García D., Andaluz-Ojeda D., Mulero M.D.R., Rodríguez-Borja E., Ballesteros-Vizoso A., Calvo M.D., Albert-Botella L., Giráldez A.P. (2022). Circulating Levels of Calprotectin, a Signature of Neutrophil Activation in Prediction of Severe Respiratory Failure in COVID-19 Patients: A Multicenter, Prospective Study (CalCov Study). Inflamm. Res..

[31] Theilgaard-Mönch K., Jacobsen L.C., Nielsen M.J., Rasmussen T., Udby L., Gharib M., Arkwright P.D., Gombart A.F., Calafat J., Moestrup S.K. (2006). Haptoglobin Is Synthesized during Granulocyte Differentiation, Stored in Specific Granules, and Released by Neutrophils in Response to Activation. Blood.

[32] Kessel C., Koné-Paut I., Tellier S., Belot A., Masjosthusmann K., Wittkowski H., Fuehner S., Rossi-Semerano L., Dusser P., Marie I. (2022). An Immunological Axis Involving Interleukin 1β and Leucine-Rich-A2-Glycoprotein Reflects Therapeutic Response of Children with Kawasaki Disease: Implications from the KAWAKINRA Trial. J. Clin. Immunol..

[33] Korkmaz B., Moreau T., Gauthier F. (2008). Neutrophil Elastase, Proteinase 3 and Cathepsin G: Physicochemical Properties, Activity and Physiopathological Functions. Biochimie.

[34] Caccavo D., Garzia P., Sebastiani G.D., Ferri G.M., Galluzzo S., Vadacca M., Rigon A., Afeltra A., Amoroso A. (2003). Expression of Lactoferrin on Neutrophil Granulocytes from Synovial Fluid and Peripheral Blood of Patients with Rheumatoid Arthritis. J. Rheumatol..

[35] Read C.B., Kuijper J.L., Hjorth S.A., Heipel M.D., Tang X., Fleetwood A.J., Dantzler J.L., Grell S.N., Kastrup J., Wang C. (2015). Cutting Edge: Identification of Neutrophil PGLYRP1 as a Ligand for TREM-1. J. Immunol..

[36] Lau D., Mollnau H., Eiserich J.P., Freeman B.A., Daiber A., Gehling U.M., Brümmer J., Rudolph V., Münzel T., Heitzer T. (2005). Myeloperoxidase Mediates Neutrophil Activation by Association with CD11b/CD18 Integrins. Proc. Natl. Acad. Sci. USA.

[37] Sarr D., Oliveira L.J., Russ B.N., Owino S.O., Middii J.D., Mwalimu S., Ambasa L., Almutairi F., Vulule J., Rada B. (2021). Myeloperoxidase and Other Markers of Neutrophil Activation Associate with Malaria and Malaria/HIV Coinfection in the Human Placenta. Front. Immunol..

[38] Scherlinger M., Richez C., Tsokos G.C., Boilard E., Blanco P. (2023). The Role of Platelets in Immune-Mediated Inflammatory Diseases. Nat. Rev. Immunol..

[39] Yamada T., Steinz M.M., Kenne E., Lanner J.T. (2017). Muscle Weakness in Rheumatoid Arthritis: The Role of Ca^2+^ and Free Radical Signaling. eBioMedicine.

[40] Donovan M.K.R., Huang Y., Blume J.E., Wang J., Hornburg D., Ferdosi S., Mohtashemi I., Kim S., Ko M., Benz R.W. (2023). Functionally Distinct BMP1 Isoforms Show an Opposite Pattern of Abundance in Plasma from Non-Small Cell Lung Cancer Subjects and Controls. PLoS ONE.

[41] Xu L., Zheng H. (2016). The Isoform II of SRSF1: A Potential Biomarker in the Progression of Pediatric Acute Lymphoblastic Leukemia. Blood.

[42] Ibáñez-Costa A., Perez-Sanchez C., Patiño-Trives A.M., Luque-Tevar M., Font P., de la Rosa I.A., Roman-Rodriguez C., Abalos-Aguilera M.C., Conde C., Gonzalez A. (2022). Splicing Machinery Is Impaired in Rheumatoid Arthritis, Associated with Disease Activity and Modulated by Anti-TNF Therapy. Ann. Rheum. Dis..

[43] Zhang F., Wei K., Slowikowski K., Fonseka C.Y., Rao D.A., Kelly S., Goodman S.M., Tabechian D., Hughes L.B., Salomon-Escoto K. (2019). Defining Inflammatory Cell States in Rheumatoid Arthritis Joint Synovial Tissues by Integrating Single-Cell Transcriptomics and Mass Cytometry. Nat. Immunol..

[44] Hu C., Dai Z., Xu J., Zhao L., Xu Y., Li M., Yu J., Zhang L., Deng H., Liu L. (2022). Proteome Profiling Identifies Serum Biomarkers in Rheumatoid Arthritis. Front. Immunol..

[45] Cheng Y., Chen Y., Sun X., Li Y., Huang C., Deng H., Li Z. (2014). Identification of Potential Serum Biomarkers for Rheumatoid Arthritis by High-Resolution Quantitative Proteomic Analysis. Inflammation.

[46] Liu S., Guo Y., Lu L., Lu J., Ke M., Xu T., Lu Y., Chen W., Wang J., Kong D. (2020). Fibrinogen-Like Protein 1 Is a Novel Biomarker for Predicting Disease Activity and Prognosis of Rheumatoid Arthritis. Front. Immunol..

[47] Mun S., Lee J., Lim M.-K., Lee Y.-R., Ihm C., Lee S.H., Kang H.-G. (2018). Development of a Novel Diagnostic Biomarker Set for Rheumatoid Arthritis Using a Proteomics Approach. BioMed Res. Int..

[48] Hayashi J., Kihara M., Kato H., Nishimura T. (2015). A Proteomic Profile of Synoviocyte Lesions Microdissected from Formalin-Fixed Paraffin-Embedded Synovial Tissues of Rheumatoid Arthritis. Clin. Proteom..

[49] Ren X., Geng M., Xu K., Lu C., Cheng Y., Kong L., Cai Y., Hou W., Lu Y., Aihaiti Y. (2021). Quantitative Proteomic Analysis of Synovial Tissue Reveals That Upregulated OLFM4 Aggravates Inflammation in Rheumatoid Arthritis. J. Proteome Res..

[50] Birkelund S., Bennike T.B., Kastaniegaard K., Lausen M., Poulsen T.B.G., Kragstrup T.W., Deleuran B.W., Christiansen G., Stensballe A. (2020). Proteomic Analysis of Synovial Fluid from Rheumatic Arthritis and Spondyloarthritis Patients. Clin. Proteom..

[51] Mateos J., Lourido L., Fernández-Puente P., Calamia V., Fernández-López C., Oreiro N., Ruiz-Romero C., Blanco F.J. (2012). Differential Protein Profiling of Synovial Fluid from Rheumatoid Arthritis and Osteoarthritis Patients Using LC–MALDI TOF/TOF. J. Proteom..

[52] Balakrishnan L., Bhattacharjee M., Ahmad S., Nirujogi R.S., Renuse S., Subbannayya Y., Marimuthu A., Srikanth S.M., Raju R., Dhillon M. (2014). Differential Proteomic Analysis of Synovial Fluid from Rheumatoid Arthritis and Osteoarthritis Patients. Clin. Proteom..

[53] Rychkov D., Neely J., Oskotsky T., Yu S., Perlmutter N., Nititham J., Carvidi A., Krueger M., Gross A., Criswell L.A. (2021). Cross-Tissue Transcriptomic Analysis Leveraging Machine Learning Approaches Identifies New Biomarkers for Rheumatoid Arthritis. Front. Immunol..

[54] Yim A.Y.F.L., Ferrero E., Maratou K., Lewis H.D., Royal G., Tough D.F., Larminie C., Mannens M.M.A.M., Henneman P., de Jonge W.J. (2021). Novel Insights into Rheumatoid Arthritis Through Characterization of Concordant Changes in DNA Methylation and Gene Expression in Synovial Biopsies of Patients with Differing Numbers of Swollen Joints. Front. Immunol..

[55] Wittkowski H., Foell D., af Klint E., Rycke L.D., Keyser F.D., Frosch M., Ulfgren A.-K., Roth J. (2007). Effects of Intra-Articular Corticosteroids and Anti-TNF Therapy on Neutrophil Activation in Rheumatoid Arthritis. Ann. Rheum. Dis..

[56] Mourão A.F., Canhão H., Sousa E., Cascão R., da Costa J.B., de Almeida L.S., Oliveira M.E., Gomes M.M., Queiroz M.V., Fonseca J.E. (2010). From a Neutrophilic Synovial Tissue Infiltrate to a Challenging Case of Rheumatoid Arthritis. Acta Reum. Port..

[57] Wright H.L., Lyon M., Chapman E.A., Moots R.J., Edwards S.W. (2021). Rheumatoid Arthritis Synovial Fluid Neutrophils Drive Inflammation Through Production of Chemokines, Reactive Oxygen Species, and Neutrophil Extracellular Traps. Front. Immunol..

[58] Chen P., Zhou G., Lin J., Li L., Zeng Z., Chen M., Zhang S. (2020). Serum Biomarkers for Inflammatory Bowel Disease. Front. Med..

[59] Rice S.J., Belani C.P. (2022). Optimizing Data-independent Acquisition (DIA) Spectral Library Workflows for Plasma Proteomics Studies. Proteomics.

[60] Fossati A., Richards A.L., Chen K.-H., Jaganath D., Cattamanchi A., Ernst J.D., Swaney D.L. (2021). Toward Comprehensive Plasma Proteomics by Orthogonal Protease Digestion. J. Proteome Res..

[61] Distler U., Łącki M.K., Schumann S., Wanninger M., Tenzer S. (2019). Enhancing Sensitivity of Microflow-Based Bottom-Up Proteomics through Postcolumn Solvent Addition. Anal. Chem..

[62] Wang Z., Mülleder M., Batruch I., Chelur A., Textoris-Taube K., Schwecke T., Hartl J., Causon J., Castro-Perez J., Demichev V. (2022). High-Throughput Proteomics of Nanogram-Scale Samples with Zeno SWATH MS. eLife.

[63] Quehenberger O., Armando A.M., Brown A.H., Milne S.B., Myers D.S., Merrill A.H., Bandyopadhyay S., Jones K.N., Kelly S., Shaner R.L. (2010). Lipidomics Reveals a Remarkable Diversity of Lipids in Human Plasma. J. Lipid Res..

[64] Yang Y., Liu X., Shen C., Lin Y., Yang P., Qiao L. (2020). In Silico Spectral Libraries by Deep Learning Facilitate Data-Independent Acquisition Proteomics. Nat. Commun..

[65] Deutsch E.W., Omenn G.S., Sun Z., Maes M., Pernemalm M., Palaniappan K.K., Letunica N., Vandenbrouck Y., Brun V., Tao S. (2021). Advances and Utility of the Human Plasma Proteome. J. Proteome Res..

[66] Aebersold R., Agar J.N., Amster I.J., Baker M.S., Bertozzi C.R., Boja E.S., Costello C.E., Cravatt B.F., Fenselau C., Garcia B.A. (2018). How Many Human Proteoforms Are There?. Nat. Chem. Biol..

[67] Palstrøm N.B., Matthiesen R., Rasmussen L.M., Beck H.C. (2022). Recent Developments in Clinical Plasma Proteomics—Applied to Cardiovascular Research. Biomedicines.

[68] Smith L.M., Kelleher N.L., Linial M., Goodlett D., Langridge-Smith P., Goo Y.A., Safford G., Bonilla L., Kruppa G., Zubarev R. (2013). Proteoform: A Single Term Describing Protein Complexity. Nat. Methods.

[69] Smith L.M., Agar J.N., Chamot-Rooke J., Danis P.O., Ge Y., Loo J.A., Paša-Tolić L., Tsybin Y.O., Kelleher N.L., Proteomics T.C. (2021). for T.-D. The Human Proteoform Project: Defining the Human Proteome. Sci. Adv..

[70] Kim H.K., Pham M.H.C., Ko K.S., Rhee B.D., Han J. (2018). Alternative Splicing Isoforms in Health and Disease. Pflügers Arch. Eur. J. Physiol..

[71] Wang X., Codreanu S.G., Wen B., Li K., Chambers M.C., Liebler D.C., Zhang B. (2018). Detection of Proteome Diversity Resulted from Alternative Splicing Is Limited by Trypsin Cleavage Specificity*. Mol. Cell. Proteom..

[72] Gornik O., Lauc G. (2008). Glycosylation of Serum Proteins in Inflammatory Diseases. Dis. Markers.

[73] Cramer D.A.T., Franc V., Caval T., Heck A.J.R. (2022). Charting the Proteoform Landscape of Serum Proteins in Individual Donors by High-Resolution Native Mass Spectrometry. Anal. Chem..

[74] Bagdonaite I., Malaker S.A., Polasky D.A., Riley N.M., Schjoldager K., Vakhrushev S.Y., Halim A., Aoki-Kinoshita K.F., Nesvizhskii A.I., Bertozzi C.R. (2022). Glycoproteomics. Nat. Rev. Methods Prim..

[75] Melby J.A., Roberts D.S., Larson E.J., Brown K.A., Bayne E.F., Jin S., Ge Y. (2021). Novel Strategies to Address the Challenges in Top-Down Proteomics. J. Am. Soc. Mass Spectrom..

[76] Cheon D.H., Yang E.G., Lee C., Lee J.E. (2017). Low-Molecular-Weight Plasma Proteome Analysis Using Top-Down Mass Spectrometry. Methods Mol. Biol..

[77] Tiambeng T.N., Wu Z., Melby J.A., Ge Y. (2022). Size Exclusion Chromatography Strategies and MASH Explorer for Large Proteoform Characterization. Methods Mol. Biol..

[78] Ntai I., Fornelli L., DeHart C.J., Hutton J.E., Doubleday P.F., LeDuc R.D., van Nispen A.J., Fellers R.T., Whiteley G., Boja E.S. (2018). Precise Characterization of KRAS4b Proteoforms in Human Colorectal Cells and Tumors Reveals Mutation/Modification Crosstalk. Proc. Natl. Acad. Sci. USA.

[79] Marx V. (2021). Tools to Cut the Sweet Layer-Cake That Is Glycoproteomics. Nat. Methods.

[80] Polasky D.A., Geiszler D.J., Yu F., Li K., Teo G.C., Nesvizhskii A.I. (2023). MSFragger-Labile: A Flexible Method to Improve Labile PTM Analysis in Proteomics. Mol. Cell. Proteom..

[81] Roberts D.S., Mann M., Melby J.A., Larson E.J., Zhu Y., Brasier A.R., Jin S., Ge Y. (2021). Structural OGlycoform Heterogeneity of the SARS-CoV-2 Spike Protein Receptor-Binding Domain Revealed by Top-Down Mass Spectrometry. J. Am. Chem. Soc..

[82] Narzo A.F.D., Brodmerkel C., Telesco S.E., Argmann C., Peters L.A., Li K., Kidd B., Dudley J., Cho J., Schadt E.E. (2019). High-Throughput Identification of the Plasma Proteomic Signature of Inflammatory Bowel Disease. J. Crohn’s Colitis.

[83] Bourgonje A.R., Hu S., Spekhorst L.M., Zhernakova D.V., Vila A.V., Li Y., Voskuil M.D., van Berkel L.A., Folly B.B., Charrout M. (2021). The Effect of Phenotype and Genotype on the Plasma Proteome in Patients with Inflammatory Bowel Disease. J. Crohn’s Colitis.

[84] Meuwis M.-A., Fillet M., Geurts P., de Seny D., Lutteri L., Chapelle J.-P., Bours V., Wehenkel L., Belaiche J., Malaise M. (2007). Biomarker Discovery for Inflammatory Bowel Disease, Using Proteomic Serum Profiling. Biochem. Pharmacol..

[85] Zhang F., Xu C., Ning L., Hu F., Shan G., Chen H., Yang M., Chen W., Yu J., Xu G. (2016). Exploration of Serum Proteomic Profiling and Diagnostic Model That Differentiate Crohn’s Disease and Intestinal Tuberculosis. PLoS ONE.

[86] (2022). The Role of Fibronectin and Its Isoforms in the Pathogenesis and Progression of Rheumatoid Arthritis: A Review. Biointerface Res. Appl. Chem..

[87] Yang C., Wang C., Zhou J., Liang Q., He F., Li F., Li Y., Chen J., Zhang F., Han C. (2020). Fibronectin 1 Activates WNT/β-Catenin Signaling to Induce Osteogenic Differentiation via Integrin Β1 Interaction. Lab. Investig..

[88] Katz D.H., Robbins J.M., Deng S., Tahir U.A., Bick A.G., Pampana A., Yu Z., Ngo D., Benson M.D., Chen Z.-Z. (2022). Proteomic Profiling Platforms Head-to-Head: Leveraging Genetics and Clinical Traits to Compare Aptamer- and Antibody-Based Methods. Sci. Adv..

[89] Huang T., Wang J., Stukalov A., Donovan M., Ferdosi S., Williamson L., Just S., Astro G., Elgierari E., Benz R. Functionalized Nanoparticles Enable Quantitative and Precise Large-Scale Unbiased, Deep Plasma Proteomics, WP 638, 2023 ASMS (Wednesday Poster, Poster ID: 314438). https://www.abstracts.asms.org/pages/dashboard.html#/conference/297/toc/297/details.

